# Preserved Function of Endothelial Colony-Forming Cells in Female Rats with Intrauterine Growth Restriction: Protection Against Arterial Hypertension and Arterial Stiffness?

**DOI:** 10.3390/cells15020171

**Published:** 2026-01-17

**Authors:** Thea Chevalley, Floriane Bertholet, Marion Dübi, Maria Serena Merli, Mélanie Charmoy, Sybil Bron, Manon Allouche, Alexandre Sarre, Nicole Sekarski, Stéphanie Simoncini, Patrick Taffé, Umberto Simeoni, Catherine Yzydorczyk

**Affiliations:** 1 DOHaD Laboratory, Department Woman-Mother-Child, Division of Pediatrics, Lausanne University Hospital and University of Lausanne, 1011 Lausanne, Switzerland; thea.chevalley@chuv.ch (T.C.); floriane.bertholet@unil.ch (F.B.); marion.dubi@unil.ch (M.D.); mariaserena.merli@gmail.com (M.S.M.); manon.allouche@chuv.ch (M.A.); alexandre.sarre@chuv.ch (A.S.); umberto.simeoni@unil.ch (U.S.); 2 Department of Oncology UNIL CHUV, University of Lausanne, 1011 Lausanne, Switzerland; melanie.charmoy@unil.ch (M.C.); sybil.bron@unil.ch (S.B.); 3 Department of Paediatric Cardiology, Lausanne University Hospital, University of Lausanne, 1011 Lausanne, Switzerland; nicole.sekarski@chuv.ch; 4 UFR de Pharmacie, Campus Santé, Centre de Recherche en Cardiovasculaire et Nutrition, Institut National de la Santé et de la Recherche Médicale, Institut National de Recherche pour l’Agriculture, l’Alimentation et l’Environnement, Aix Marseille University, 13385 Marseille, France; stephanie.simoncini@univ-amu.fr; 5 Center for Primary Care and Public Health (Unisanté), University of Lausanne, 1011 Lausanne, Switzerland; patrick.taffe@unisante.ch

**Keywords:** endothelial colony-forming cells, arterial hypertension, arterial stiffness, intrauterine growth restriction, oxidative stress, stress-induced premature senescence

## Abstract

**Highlights:**

**What are the main findings?**

- Intrauterine growth restriction (IUGR) is a risk factor for long-term vascular outcomes.
- A sexual dimorphism has been identified: compared to control, only IUGR male rats displayed vascular dysfunctions at 6 months of age.

**What are the implications of the main findings?**

- IUGR female rats show only minor changes in endothelial colony forming cells (ECFCs) functions.
- The absence of ECFC alterations could protect IUGR females against vascular dysfunctions.

**Abstract:**

Individuals born after intrauterine growth restriction (IUGR) are at increased risk of long-term cardiovascular complications, including elevated blood pressure, endothelial dysfunction, and arterial stiffness. Endothelial progenitor cells (EPCs), particularly endothelial colony-forming cells (ECFCs), play a critical role in maintaining vascular homeostasis. Previously, Simoncini et al. observed that in a rat model of IUGR, six-month-old males exhibited elevated systolic blood pressure (SBP) and microvascular rarefaction compared with control (CTRL) rats. These vascular alterations were accompanied by reduced numbers and impaired function of bone marrow-derived ECFCs, which were associated with oxidative stress and stress-induced premature senescence (SIPS). In contrast, IUGR females of the same age and from the same litter did not exhibit higher SBP or microvascular rarefaction, raising the question of whether ECFC dysfunction in IUGR female rats can be present without vascular alterations. So, we investigated ECFCs isolated from six-month-old female IUGR offspring (maternal 9% casein diet) and CTRL females (23% casein diet). To complete the vascular assessment, we performed in vivo and in vitro investigations. No alteration in pulse wave velocity (measured by echo-Doppler) was observed; however, IUGR females showed decreased aortic collagen and increased elastin content compared with CTRL. Regarding ECFCs, those from IUGR females maintained their endothelial identity (CD31^+^/CD146^+^ ratio among viable CD45^−^ cells) but exhibited slight alterations in progenitor marker expression (CD34) compared with those of CTRL females. Functionally, IUGR-ECFCs displayed a delayed proliferation phase between 6 and 24 h, while their ability to form capillary-like structures remained unchanged, however their capacity to form capillary-like structures was preserved. Regarding the nitric oxide (NO) pathway, a biologically relevant trend toward reduced NO levels and decreased endothelial nitric oxide synthase expression was observed, whereas oxidative stress and SIPS markers remained unchanged. Overall, these findings indicate that ECFCs from six-month-old female IUGR rats exhibit only minor functional alterations, which may contribute to vascular protection against increase SBP, microvascular rarefaction, and arterial stiffness.

## 1. Introduction

Endothelial dysfunction, arterial stiffness, and arterial hypertension (AHT) are closely interconnected components of a self-perpetuating cycle that progressively damages the vascular system, as demonstrated in both human and animal studies [1,2,3]. Endothelial progenitor cells (EPCs) play an important role in endothelial homeostasis. EPCs can be isolated predominantly from umbilical cord blood, bone marrow, and peripheral circulation and can be categorized according to their phenotype [4,5]. Early-stage EPCs contribute to angiogenesis through paracrine mechanisms; these cells cannot develop into mature endothelial cells. In contrast, late-growing EPCs, or endothelial colony-forming cells (ECFCs), exhibit clonal potential with the capacity to produce mature endothelial cells [6,7]. ECFCs can proliferate, migrate, differentiate, self-renew, and promote vascular growth and neovascularization in vitro and in vivo [8]. For instance, in neonatal mice subjected to hyperoxia, administration of human ECFCs promoted their direct incorporation into the injured vascular network, thereby markedly attenuating hyperoxia-induced avascular regions, enhancing physiological vascularization, and preventing the development of pathological preretinal neovascularization [9]. ECFCs are identified by the presence of surface markers, such as CD34 [10] and vascular endothelial growth factor receptor-2 (VEGFR-2, also named KDR) [11], however do not express the hematopoietic marker CD45 [12]. A reduced number or impaired function has been demonstrated to be a key mechanism linking endothelial dysfunction to the pathogenesis of cardiovascular diseases (CVD) [13,14,15]. The colony count of EPCs has been inversely correlated with systolic and diastolic blood pressure in hypertensive subjects [16]. In the early stages of AHT, reduced numbers of circulating EPCs have been observed and this measure may represent a reliable predictor of endothelial dysfunction as well as a marker of target organ damage [17,18], both functional and structural [19]. In addition, a positive correlation has been observed between the number of circulating EPCs and arterial stiffness, suggesting a role in arterial remodeling [20].

Individuals born with low birth weight, either due to premature birth or intrauterine growth restriction (IUGR), have an increased risk of developing CVD later in life [21]. This includes conditions such as AHT [22,23] and arterial stiffness, which are associated with impaired elastin synthesis in the walls of the aorta and large arteries and may lead to permanent changes in their mechanical properties [24,25]. In addition, endothelial dysfunction has been demonstrated in full-term infants, children, and young adults born after IUGR [26,27]. Bertagnolli et al., have shown that adults born very preterm have impaired ECFC functionality, compared with term controls associated with elevated systolic blood pressure (SBP) [28]. In previous work, we were able to demonstrated that, from birth, ECFCs from growth-restricted newborns were reduced in number and had altered angiogenic properties linked to stress-induced premature senescence (SIPS) [29]. Several studies have reported an association of fetal growth restriction with cellular senescence and oxidative stress [30,31], as well as a negative impact on EPCs [32,33] in humans [34] and animal models [35]. In a rat model, Simoncini et al. [36] demonstrated that ECFCs isolated from the bone marrow of six-month-old IUGR males were reduced in number and had impaired function, associated with increased SBP and microvascular rarefaction, compared to ECFCs from control (CTRL) male animals. Indeed, the ECFCs had altered proliferative capacity and ability to form vascular networks, as well as reduced production of nitric oxide (NO), a key factor in angiogenesis. These dysfunctions have been linked to the presence of markers related to oxidative stress and SIPS. However, IUGR females of the same age and from the same litter than males did not have higher SBP or microvascular rarefaction, prompting the question as to whether ECFC dysfunction in female IUGR rats can be present without vascular alterations.

In this study, we extended the vascular exploration in CTRL and IUGR females by studying arterial stiffness in vivo and in vitro, followed by an assessment to determine whether IUGR alters the phenotype, the proliferative capacity, and the vascular network formation capacity of ECFCs. In addition, we evaluated the angiogenic potential of ECFCs and the expression of molecular markers related to oxidative stress and SIPS.

## 2. Materials and Methods

### 2.1. Animal Model

We developed a rat model of IUGR by randomly assigning pregnant rats to either a CTRL diet containing 23% casein (CTRL group; version 0001 210, SAFE, Augy, France) or an isocaloric low-protein diet containing 9% casein (IUGR group; version 0040, SAFE). All procedures were conducted in accordance with the Swiss Veterinarian Animal Care Office and were approved by the ethics committee for animal research at the University of Lausanne (VD3050), as previously described [37]. We have previously shown that pups in the IUGR group exhibited a lower birth weight than those in the CTRL group, a difference that persisted into adulthood, at six months of age [36]. Litter sizes were standardized to ten pups to ensure uniform nutritional intake until weaning; thereafter, animals in both groups had free access to a standard diet (A04, SAFE Diets, Augy, France) and water. In the present study, each animal originated from a different litter rats were euthanized at six months of age by intraperitoneal injection of pentobarbital (Esconarkon, Streuli Pharma AG; 150 mg/kg body weight), followed by blood collection.

### 2.2. Assessment of Pulse Wave Velocity (PWV)

Rats of 6 months of age were lightly anesthetized with isoflurane (in O_2_) in order to maintain heat rate at 400–450 bpm. The animals were then placed on a warming pad to maintain normothermia in a prone position. The chest and abdominal hair were shaved, and the paws were attached on pad electrodes allowing electrocardiography (ECG) recording. Using a MX250 probe (13–24 MHz) and Vevo 3100 ultrasound machine (VisualSonics, Toronto, ON, Canada), Doppler tracings of aortic outflow tract and descending abdominal aorta, immediately above the aortic bifurcation, were recorded simultaneously with the ECG. All Doppler measurements were obtained within 5–10 min after the anesthesia induction; the animals were then allowed to recover from the anesthesia and placed back in their cage [38]. Each tracing was then reviewed with Visualsonics software. The time elapsed from the onset of the R wave of the QRS to the onset of the flow wave was measured. For each tracing, at least three measures were performed. For each rat, a time to the onset of the aortic valve (T1) and to the aortic bifurcation (T2) was obtained. The difference corresponds to the transit time of the pulse wave from the heart to the aortic bifurcation. The heart-bifurcation transit distance was measured from body surface points, which is the traditional body surface method and was commonly used [39]. PWV was calculated by dividing transit distance by transit time.

### 2.3. Arterial Stiffness Evaluation

Thoracic aortas were dissected, fixed in 10% neutral buffered formalin and paraffin embedded. Sequential 5 μm sections were stained with Van Gieson Elastic Lamina or Masson’s trichrome stains for elastin and collagen fibers, respectively. On the histological sections, the proportion of elastin and density fibers in media area were quantified using ImageJ software (Java 1.8.0_112, National Institutes of Health, Southern Montgomery, USA, accessed 1 July 2021) [40,41]. Images were acquired using an Eclipse Ti2 fluorescence microscope (Nikon Europe B.V., Amsterdam, the Netherlands) at 5× magnification and analyzed by a single investigator (T.C.) blinded.

### 2.4. Isolation of ECFCs

ECFCs were isolated from bone marrow as previously described by Simoncini et al. [36]. Bone marrow mononuclear cells (MNCs) were obtained from six-month-old female CTRL and IUGR rats using density gradient centrifugation with Histopaque 1077 (Sigma-Aldrich, Saint Louis, MO, USA). After centrifugation, MNCs were washed in RPMI medium supplemented with 10% fetal calf serum (FCS; Thermo Fisher Scientific, Rockford, IL, USA) and resuspended in endothelial basal medium-2 (EBM-2) supplemented with endothelial cell growth medium MV2 (PromoCell, Heidelberg, Germany) and penicillin/streptomycin (Sigma-Aldrich). Cells were seeded onto a gelatin-coated surface (0.2%). Gelatin matrices have been shown to provide a favorable environment for ECFC culture, supporting both proliferation and vasculogenic potential [42,43]. ECFC colonies were identified by their characteristic cobblestone morphology using an inverted microscope (Eclipse Ti2 Series, Nikon), and non-cobblestone cells were removed. ECFCs were used for experiments between passages 1 and 3 after isolation. Each ECFC culture corresponded to an individual animal from a separate litter. Some ECFC cultures failed to expand due to cell fragility or contamination; these failures occurred in both groups without an apparent pattern. Although a slight selection bias cannot be entirely excluded, the limited number of successful cultures is more likely to affect statistical power than to introduce systematic bias, which accounts for the differences in sample size across experiments. The female ECFCs analyzed in this study were derived from the same litters as the previously published male ECFCs [36] and were isolated and cultured under identical conditions. Although the female experiments were not conducted simultaneously with the male studies, the shared origin and standardized handling support the validity of indirect comparisons between sexes.

### 2.5. ECFC Quantification Using Flow Cytometry

Single-cell suspensions of ECFCs from CTRL (n = 4) and IUGR (n = 4) rats were stained with fluorochrome-labeled monoclonal antibodies against CD31 PE (TLD-3A12), CD45 FITC (OX-1), and CD146 APC (LSEC) (BD Biosciences, San Jose, CA, USA or Miltenyi Biotech, Bergisch Gladbach, Germany) in phosphate-buffered saline (PBS; CHUV) containing 3% fetal calf serum (FCS) for 20 min at 4 °C. After removal of unbound antibodies by centrifugation, cells were resuspended in 200 µL of PBS/3%FCS, and 0.5 µg of 4′,6-diamidino-2-phenylindole (DAPI; Thermo Fisher Scientific) was added to identify dead cells. Samples were analyzed using a LSRII SORP flow cytometer equipped with 5 lasers (BD Biosciences), and data were analyzed using FlowJo software (FlowJo v10.8, Ashland, OR, USA).

### 2.6. CD34 Expression Measurement by Immunofluorescence

ECFCs from CTRL (n = 4) and IUGR (n = 4) rats were cultured in 12-well plates with gelatin-coated coverslip (0.2%). This allows an optimal cell adhesion and better preserves their morphology and membrane integrity, which facilitates the recognition of surface antigens during immunofluorescence staining. Cells were washed with PBS and fixed with cold 70% ethanol at room temperature. After PBS washes, membrane permeabilization was performed using PBS containing 0.1% Triton (AppliChem, Darmstadt, Germany), followed by blocking with PBS containing 3% Bovine Serum Albumin (BSA; (AppliChem) for 2 h at room temperature. Cells were incubated overnight at 4 °C with the primary antibody (CD34; rabbit; Cell Signaling Technology, Danvers, MA, USA) diluted 1:100 in PBS containing 0.1% Triton and 3% BSA. The next day, cells were washed with PBS and incubated for 2 h at room temperature in the dark with Alexa Fluor 488-conjugated goat anti-rabbit IgG (Cell Signaling) diluted 1:200 in PBS containing 0.1% Triton and 3% BSA. After final PBS washes, slides were mounted with Fluoromount-G containing DAPI (Interchim, Montluçon, France). A negative control was included using only the secondary antibody. Images were acquired by the same investigator (T.C.) blinded to sample identity using a fluorescence microscope (Eclipse Ti2 Series) at 20× magnification, with consistent exposure settings. Fluorescence was normalized to DAPI fluorescence using ImageJ and autofluorescence was subtracted. Fluorescence intensity was quantified and expressed as mean ± SEM. Experiment was performed in duplicate [36].

### 2.7. ECFC Proliferation Test

The proliferative capacity of ECFCs (20,000 cells/well) was assessed by measuring DNA synthesis at 6 and 24 h in CTRL (n = 4–5) and IUGR (n = 5) rats using a colorimetric cell proliferation ELISA based on the incorporation of bromodeoxyuridine (BrdU; Roche Diagnostics, Basel, Switzerland) during DNA replication. Absorbance was measured at 450 nm. Each experiment was performed in quadruplicate [12].

### 2.8. Capillary-like Structure Formation

The ability of ECFCs to form capillary-like structures was evaluated using Matrigel™ BD Growth Factor (BD Biosciences) in 96-well plates in CTRL (n = 4) and IUGR (n = 4–5) rats [36]. Matrigel was thawed on wet ice, while sterile tips were kept at −20 °C and pipettes and Eppendorf tubes at 4 °C. Matrigel was added to each well. Then, plates were incubated at 37 °C for 30 min before seeding ECFCs (20,000 cells/well). Each experiment was performed in triplicate. Images were captured at 6 and 24 h using an inverted microscope (Nikon Eclipse Ti2) at 5× magnification [12].

### 2.9. Measurement of NO Production

NO production in ECFCs from CTRL (n = 5) and IUGR (n = 5) rats was detected using the NO-specific fluorescent dye 5,6-Diaminofluorescein diacetate (DAF-2DA) (10 µM; Sigma-Aldrich) and incubated at 37 °C for 1 h in a light-protected, humidified chamber. Cells were then incubated in 4-(2-hydroxyethyl)-1-piperazineethanesulfonic acid (HEPES) buffer (Sigma-Aldrich), alone or with acetylcholine (Sigma-Aldrich; 100 µM), for another hour. Samples were mounted with Fluoromount-G containing DAPI (Interchim) and analyzed using a fluorescence microscope (Eclipse Ti2 Series) by an investigator (T.C.) blinded to sample identity. Two images were captured per well using standardized exposure settings, at 20× magnification. DAF-2DA fluorescence (expressed as mean ± SEM) was normalized to DAPI fluorescence, autofluorescence was subtracted, and images were analyzed thanks to ImageJ. Experiments were performed in duplicate [44].

### 2.10. Measurement of Superoxide Anion Production

Superoxide anion (•O_2_^−^) production was assessed in ECFCs isolated from CTRL (n = 4) and IUGR (n = 5) rats using the fluorescent probe dihydroethidium (DHE; 2 µM; Sigma-Aldrich) [31]. DHE is oxidized in the presence of superoxide anions to form ethidium bromide, which intercalates into DNA and emits red fluorescence. After incubation, coverslips were mounted using Fluoromount-G containing DAPI (Interchim). Negative controls consisted of ECFCs incubated without DHE. Fluorescence images were acquired using an Eclipse Ti2 fluorescence microscope (Nikon) at 20× magnification and analyzed by a single investigator (T.C.) blinded. Superoxide-dependent fluorescence intensity, expressed as mean ± SEM, was quantified using ImageJ software, normalized to DAPI fluorescence, and corrected for autofluorescence. Data are all experiments were performed in duplicate [44].

### 2.11. Protein Expression by Western Blotting

Proteins were extracted from ECFCs obtained from CTRL (*n* = 5) and IUGR (*n* = 4) rats using a lysis buffer as previously described [36]. Protein samples were denatured with NuPAGE reducing buffer (Life Technologies Europe B.V., Zug, Switzerland) at 70 °C for 10 min, and 35 μg of total protein per sample was resolved on NuPAGE 4–12% Bis-Tris gradient gels (Life Technologies), then transferred overnight onto nitrocellulose membranes (Whatman, Life Technologies) at 30 V and 4 °C. Successful transfer was verified by Ponceau S staining (Life Technologies).

Membranes were blocked for 2 h at room temperature in PBS containing Tween-20 (PBST; Sigma-Aldrich) supplemented with 2% (BSA). They were then incubated overnight at 4 °C with primary antibodies against Sirtuin-1, endothelial nitric oxide synthase (eNOS), Cu/Zn superoxide dismutase (SOD1), catalase, and β-actin, all diluted 1:1000 according to the manufacturer’s recommendations and prior optimization. After washes in PBST (10 min each), membranes were incubated for 2 h at room temperature with a goat anti-rabbit secondary antibody (1:1000) in blocking buffer. Following additional PBST washes, immunoreactive bands were visualized using an enhanced chemiluminescence substrate (Life Technologies) and detected with a G-BOX imaging system (GeneSys, Syngene, Cambridge, UK). Band intensities were quantified using ImageJ software and normalized to β-actin. To preserve the biological material, eNOS and Catalase were incubated on the same membrane (hence the same actin), and SOD1 and Sirtuin-1 were incubated on another membrane.

### 2.12. Senescence Detection in ECFCs

The activity of β-galactosidase (β-gal), a key characteristic of senescent cells, was assessed in ECFCs from CTRL (n = 4) and IUGR (n = 4) rats using a detection kit (Cell Signaling Technology), in accordance with the manufacturer’s instructions. β-galactosidase catalyzes the hydrolysis of X-Gal with optimal activity at pH = 6, yielding a blue reaction product detectable in senescent cells [45]. For each sample, an average of eight images were acquired using an inverted microscope (Nikon Eclipse Ti2, Nikon) at 20× magnification. The proportion of β-gal-positive cells was calculated relative to the total number of cells [29].

### 2.13. Statistical Analyses

All data are presented as mean ± SEM. We used GraphPad Prism 8 (version 8.3.0 (538), La Jolla, CA, USA) for graphical representation, and statistical analyses were carried out using Stata 18 statistical software. For cross-sectional data, the statistical comparisons were performed using the two-sided and one-sided Student *t*-test (when substantial knowledge allowed us to restrict the critical region of the test), and linear mixed-effects models for the longitudinal designs (i.e., with two repeated measurements per subject, over the two time periods). Given the very small sample sizes, no normality tests were done, and the selection of the most appropriate Student *t*-test with equal or unequal variances was simply based upon inspection of the spread of the points on the dot plots [46,47]. Statistical significance was set at *p* ≤ 0.05.

## 3. Results

### 3.1. Assessment of Arterial Stiffness

**Figure 1:**
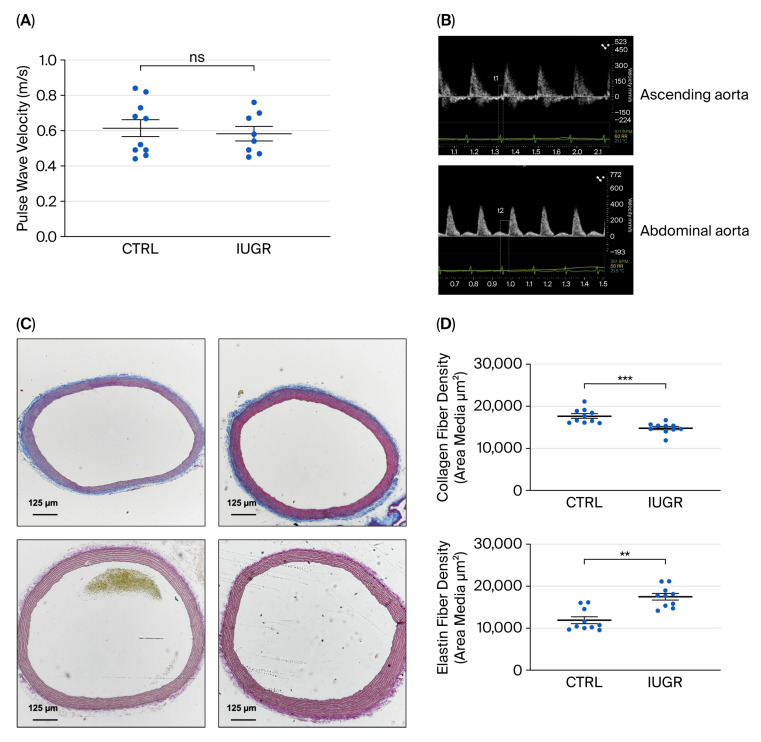
Assessment of arterial stiffness. (**A**) Measurement of PWV using Doppler ultrasound in 6-month-old CTRL (n = 10) and IUGR (n = 8) female rats. (**B**) Representative Doppler images of measurement of the time elapsed from the onset of R wave (ECG) to the aortic outflow tract (top, T1) and the abdominal aorta bifurcation (bottom, T2). (**C**) These pictures are representative images from collagen and elastin content in the aorta of 6-month-old CTRL and IUGR female rats (n = 10 per group) using Masson’s Trichrome and Verhoeff’s Van Gieson Elastic Lamina staining, respectively. Scale bar = 100 μm. (**D**) Quantification of collagen and elastin fibers density. ** *p* < 0.01; *** *p* < 0.001; ns: not significant.

PWV analysis showed no significant difference between CTRL (n = 10) and IUGR (n = 8) females (mean ± SEM, m/s: 0.614 ± 0.134 vs. 0.583 ± 0.096; *p* = 0.779; Figure 1A). We next evaluated the collagen and elastin content in the aortas. Collagen fiber density was significantly reduced in IUGR (n = 10) compared to CTRL (n = 10) females (mean ± SEM, μm^2^: 14,813 ± 880 vs. 17,670 ± 1399; *p* = 0.0002), whereas elastin fiber density was significantly increased in IUGR (n = 10) compared to CTRL (n = 10) females (mean ± SEM, μm^2^: 17,493 ± 1976 vs. 11,905 ± 2214; *p* = 0.001) (Figure 1C,D).

### 3.2. ECFC Quantification and Phenotyping

**Figure 2:**
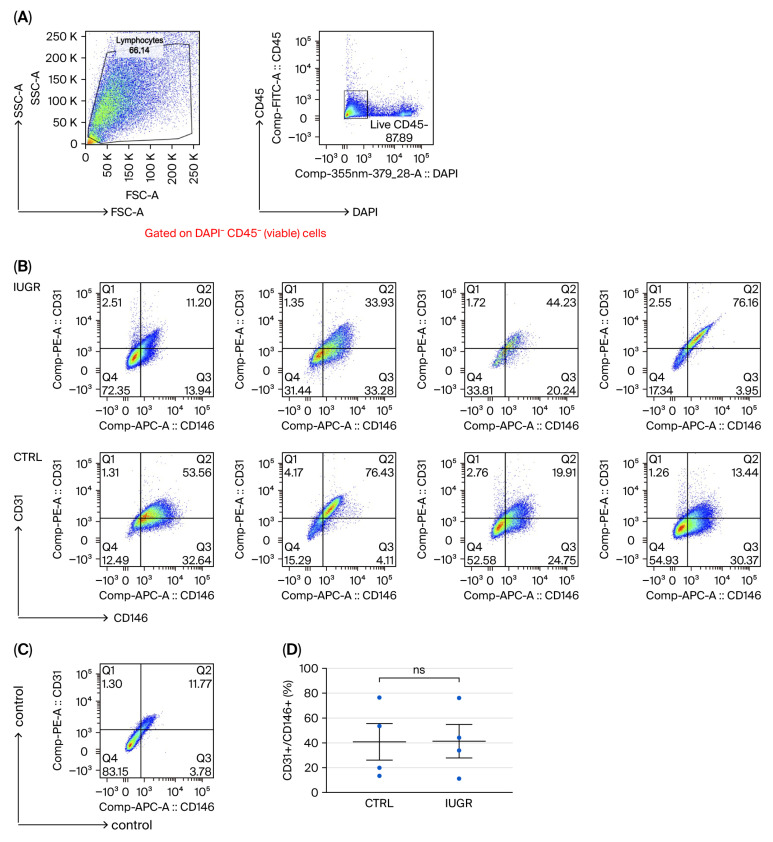
ECFC quantification. Flow cytometry analysis of cultured cells was performed on ECFCs isolated from six-month-old female CTRL and IUGR rats. (**A**) Left panel: forward scatter (FSC) versus side scatter (SSC) plot. Cells were gated to exclude subcellular debris; right panel: CD45 versus 4′,6-diamidino-2-phenylindole (DAPI) staining on gated cells from the left panel. Dead cells (DAPI^+^) and hematopoietic cells (CD45^+^) were excluded by gating on CD45-viable cells and the ratio of CD31^+^ to CD146^+^ staining was assessed. (**B**) Upper panels: cells from IUGR rats, lower panels: cells from CTRL rats; (**D**) The percentage of ECFCs CD31^+^/CD146^+^ is shown in the histogram. (**C**) Negative control stain on CD45-viable cells (in the absence of CD31 and CD146). ns: not significant.

**Figure 3:**
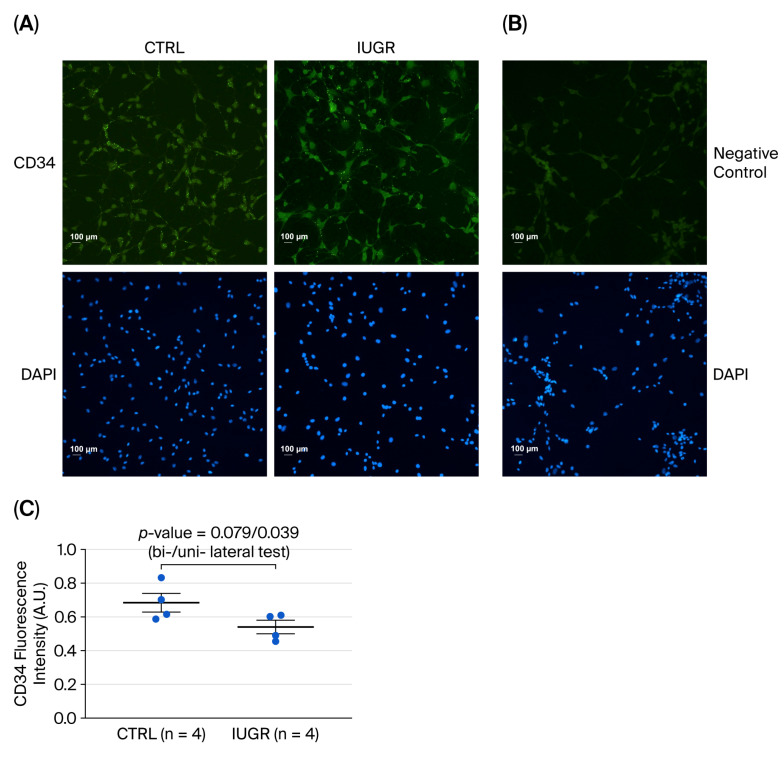
CD34 expression in ECFCs isolated from six-month-old female rats. (**A**) CD34 expression was measured by immunostaining in CTRL-ECFCs and IUGR-ECFCs. These pictures are representative images from n = 4 animals/group. Nuclei were counterstained with DAPI. (**B**) Negative control is shown. Magnification (20×). (**C**) Quantification of CD34 fluorescence intensity in ECFCs (arbitrary units, A.U.). Scale bar = 100 μm.

Flow cytometry analysis revealed no difference in the ratio of CD31^+^ to CD146^+^ staining on CD45^−^ viable ECFCs from control (CTRL, n = 4) and IUGR (n = 4) rats (mean (%) ± SEM): 40.83 ± 14.77 vs. 41.38 ± 13.49; *p* = 0.979 (bilateral Student *t*-test) (Figure 2). On the other hand, a unilateral Student *t*-test showed a statistically significant difference (*p* = 0.039) in CD34 expression between ECFCs from CTRL and IUGR rats (n = 4 for both (mean ± SEM: 0.69 ± 0.06 vs. 0.54 ± 0.04), although this was not the case for the bilateral Student *t*-test (*p* = 0.079) (Figure 3). Nevertheless, given the reduced CD34 expression found in IUGR males observed in our previous study [36], we also expected a reduced expression in IUGR female rats. Therefore, the use of a more powerful unilateral test seems warranted in our setting.

### 3.3. ECFC Function

#### 3.3.1. Proliferation Capacity

**Figure 4:**
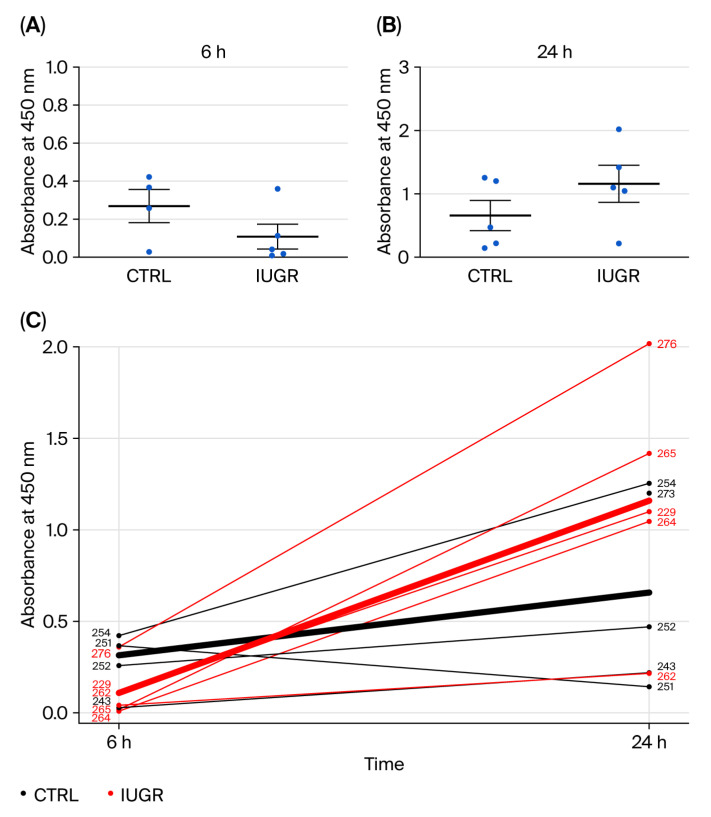
Proliferation properties of ECFCs isolated from six-month-old female rats. The proliferation capacity of CTRL-ECFCs and IUGR-ECFCs was quantified at an absorbance of 450 nm at 6 h (CTRL: n = 4 and IUGR: n = 5) (**A**) and at 24 h (n = 5/group) (**B**) after BrdU incorporation. (**C**) Represent the individual absorbance value between 6 and 24 h per sample. Each line connects paired measurements from the same sample at 6 and 24 h. Black lines represent CTRL samples, and red lines represent IUGR samples. Bold lines represent the mean time-trajectory for each group.

There was a statistically significant difference (*p* = 0.048) in the time-profiles of the proliferation capacity of ECFCs from CTRL and IUGR rats, measured first at 6 (CTRL (n = 4) vs. IUGR (n = 5) (mean (A.U.) ± SEM): 0.268 ± 0.087 vs. 0.108 ± 0.066; ) and then at 24 h (h) (CTRL (n = 5) vs. IUGR (n = 5) (mean (A.U.) ± SEM): 0.657 ± 0.239 vs. 1.159 ± 0.292), by measuring absorbance at 450 nm following BrdU incorporation during DNA synthesis (Figure 4).

#### 3.3.2. Capillary-like Outgrowth Development

**Figure 5:**
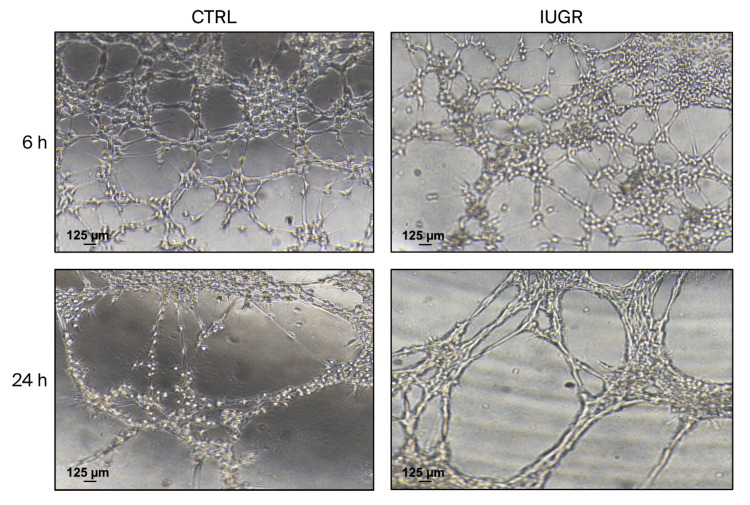
Capillary-like structure formation capacity of ECFCs isolated from six-month-old female rats. The capillary-like outgrowth sprouts of CTRL-ECFCs and IUGR-ECFCs were assessed using Matrigel cultures at 6 and 24 h (h) after seeding. Magnification (5×). These pictures are representative images from each group at 6 h (n = 4/group) and 24 h (CTRL: n = 4 and IUGR: n = 5). Scale bar = 125 μm.

There was no statistically significant difference between ECFCs from CTRL and IUGR rats in the formation of capillary-like structures. ECFCs from both groups could form endothelial networks, characterized by numerous, long, closed tubes and branches, at 6 (CTRL (n = 4) vs. IUGR (n = 4)) and 24 h (CTRL (n = 4) vs. IUGR (n = 5)) when cultured on Matrigel (Figure 5).

### 3.4. NO Pathway

#### 3.4.1. NO Production

**Figure 6:**
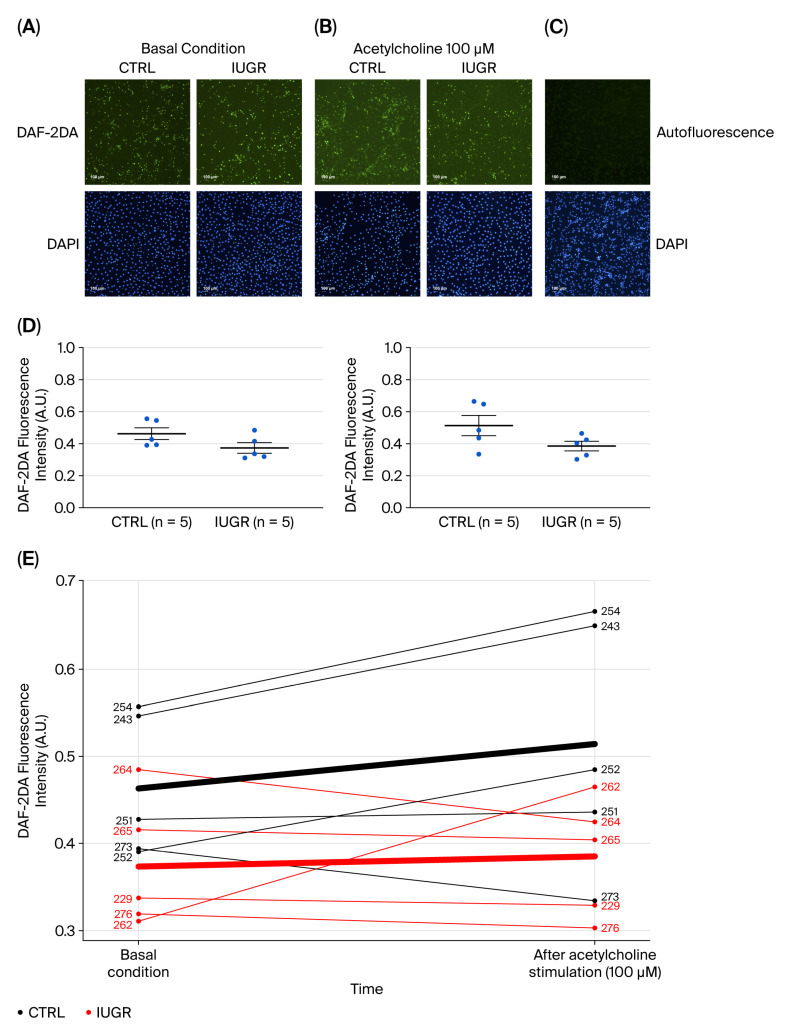
NO production in ECFCs isolated from six-month-old female rats. NO production was measured by immunostaining using DAF-2DA in CTRL-ECFCs and IUGR-ECFCs under basal conditions (using DAF-2DA only) (**A**) and after stimulation by acetylcholine (100 µM) (**B**). These pictures are representative images from n = 5 animals/group. Nuclei were counterstained with DAPI. (**C**) Autofluorescence test was also performed. Magnification (20×). Scale bar = 100 μm. (**D**) Quantification of DAF-2DA fluorescence intensity in ECFCs (arbitrary units, A.U.). (**E**) Individual values of DAF-2DA fluorescence intensity (arbitrary unit (A.U)) are shown for each sample under basal conditions and after acetylcholine stimulation. Each line connects paired measurements from the same sample before and after acetylcholine exposure. Black lines represent CTRL samples, and red lines represent IUGR samples. Bold lines represent the mean time-trajectory for each group.

Despite a borderline *p*-value (*p* = 0.060), there was an obvious difference in the time-profiles of the NO immunofluorescence expression between ECFCs from CTRL and IUGR rats measured at basal state (CTRL (n = 5) vs. IUGR (n = 5) (mean (A.U.) ± SEM): 0.46 ± 0.04 vs. 0.37 ± 0.03) and after stimulation with acetylcholine (CTRL (n = 5) vs. IUGR (n = 5) (mean (A.U.) ± SEM): 0.51 ± 0.06 vs. 0.38 ± 0.03), when using DAF-2DA specific dye (Figure 6). Because of the small sample sizes, we did not find a statistically significant comparison. Nevertheless, based on inspection of the graph and given the two clearly different time-trends, it seems justified to claim that the two groups differ in NO immunofluorescence expression.

#### 3.4.2. eNOS Expression

**Figure 7:**
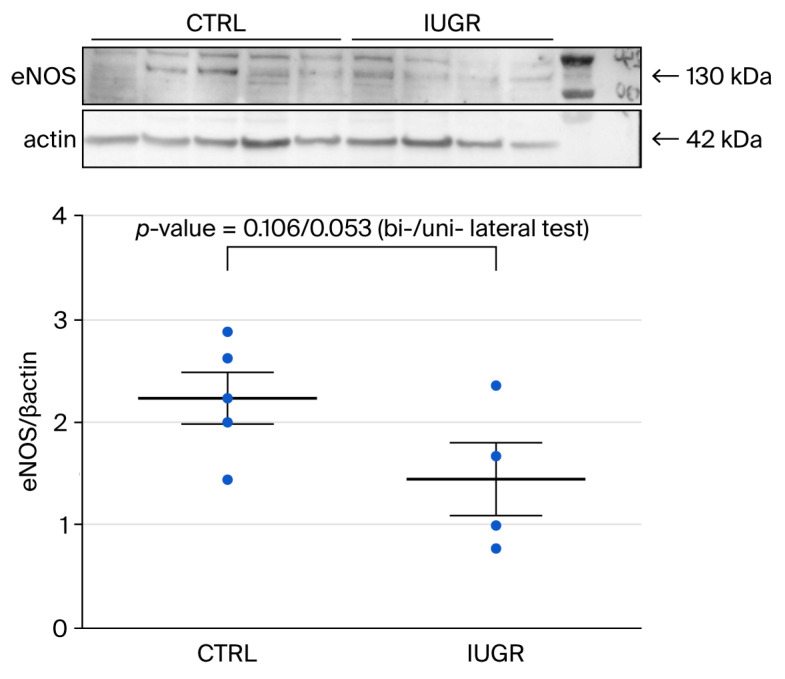
eNOS protein expression measured by Western blot in ECFCs isolated from six-month-old female CTRL (n = 5) and IUGR (n = 4) rats.

Western blot revealed a one-sided statistically significant Student *t*-test (*p* = 0.053) for the difference in eNOS expression between ECFCs from CTRL and IUGR rats (CTRL (n = 5) vs. IUGR (n = 4) (mean (A.U.) ± SEM): 2.23 ± 0.25 vs. 1.44 ± 0.36), whereas this was not the case of the two-sided Student *t*-test (*p* = 0.106) (Figure 7). Given the reduced eNOS expression found in the study of Simoncini et al. [36], the use of a more powerful unilateral test seems again warranted in our setting.

### 3.5. Processes Influencing Number and Function of ECFCs

#### 3.5.1. Oxidative Stress

##### Superoxide Anion Production

**Figure 8:**
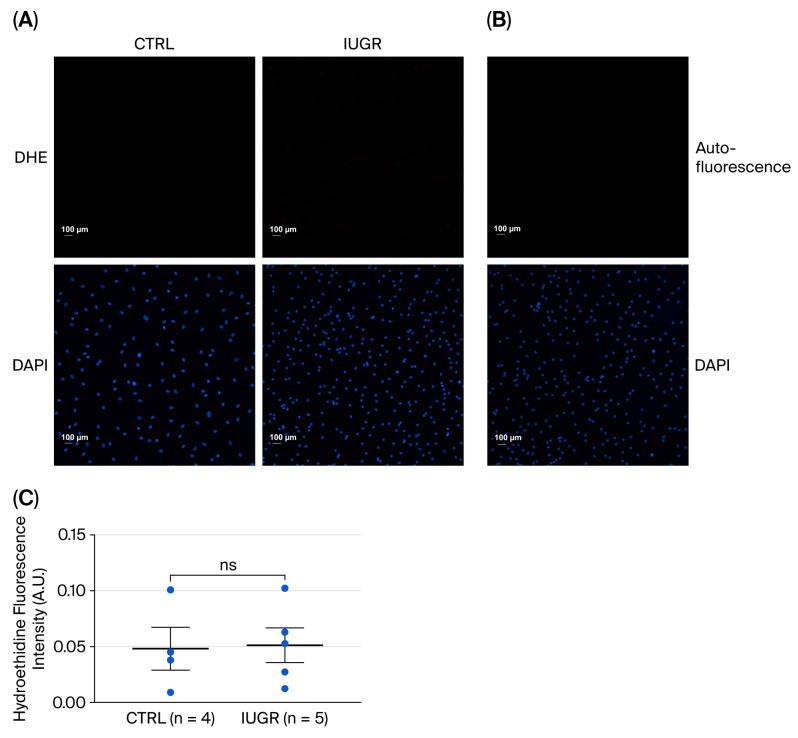
Superoxide anion production in ECFCs isolated from six-month-old female rats. (**A**) Superoxide anion production was assessed using DHE in CTRL-ECFCs and IUGR-ECFCs. These pictures are representative images from n = 4 animals (CTRL group) and n = 5 (IUGR group). Nuclei were counterstained with DAPI. (**B**) Autofluorescence test was performed. Magnification (20×). Scale bar = 100 μm. (**C**) Quantification of dihydroethidine fluorescence intensity in ECFCs (arbitrary units, A.U.). ns: not significant.

There was no statistically significant difference in superoxide anion production between ECFCs from the CTRL and IUGR rats (CTRL (n = 4) vs. IUGR (n = 5) (mean (A.U.) ± SEM): 0.048 ± 0.026 vs. 0.051 ± 0.025; *p* > 0.05) when using the oxidative fluorescent dye DHE (Figure 8).

##### Antioxidant Defenses

**Figure 9:**
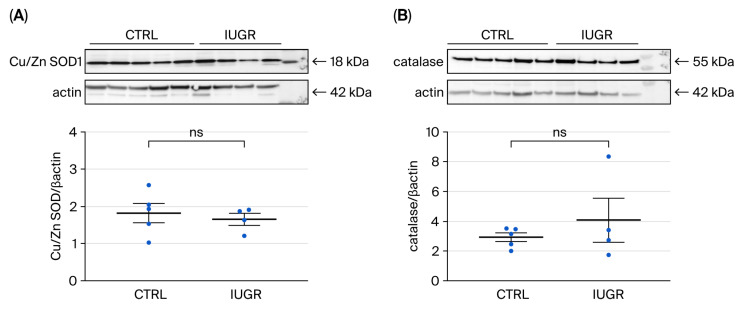
Antioxidant protein expression in ECFCs isolated from six-month-old female rats. (**A**) Cu/Zn SOD1 and (**B**) catalase protein expression were measured by Western blot in ECFCs from CTRL (n = 5) and IUGR (n = 4) rats. ns: not significant.

Western blot revealed no significant differences in Cu/Zn SOD1 expression between ECFCs from CTRL and IUGR rats (CTRL (n = 5) vs. IUGR (n = 4) (mean (A.U.) ± SEM): 1.81 ± 0.43 vs. 1.65 ± 0.23; *p* > 0.05), or in catalase expression (CTRL (n = 5) vs. IUGR (n = 4) (mean (A.U.) ± SEM): 2.91 ± 0.54 vs. 4.06 ± 2.14; *p* > 0.05) (Figure 9).

#### 3.5.2. Cellular Senescence

##### β-Galactosidase Activity

**Figure 10:**
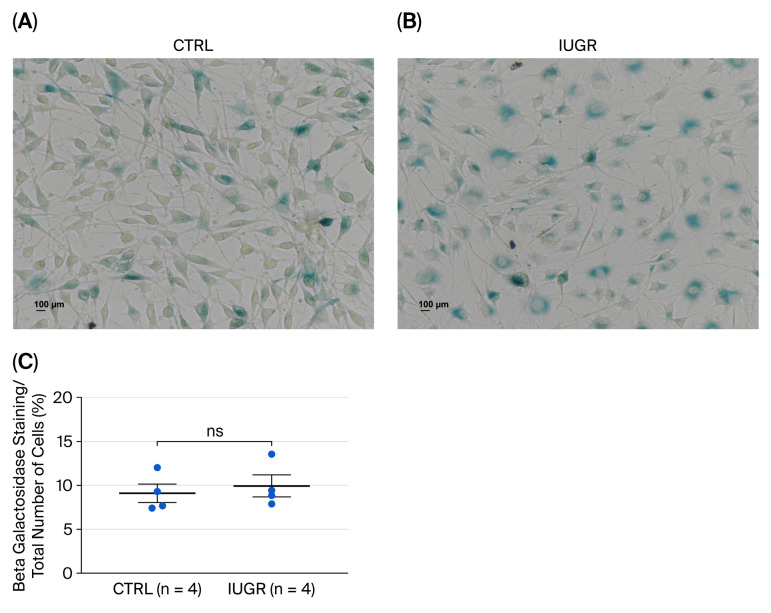
β-galactosidase activity in ECFCs isolated from six-month-old female rats. Cellular senescence was evaluated by β-gal staining in (**A**) CTRL-ECFCs and (**B**) IUGR-ECFCs. These pictures are representative images from n = 4 animals/group. Magnification (20×). Scale bar = 100 μm. (**C**) Percentage of cells positive for β-gal staining relative to the total cell number. ns: not significant.

There was no significant difference in β-gal staining between ECFCs from CTRL and IUGR rats (CTRL (n = 4) vs. IUGR (n = 4) (mean (A.U.) ± SEM): 9.10 ± 0.1.56 vs. 9.94 ± 1.82; *p* > 0.05) (Figure 10).

##### Sirtuin-1 Expression

**Figure 11:**
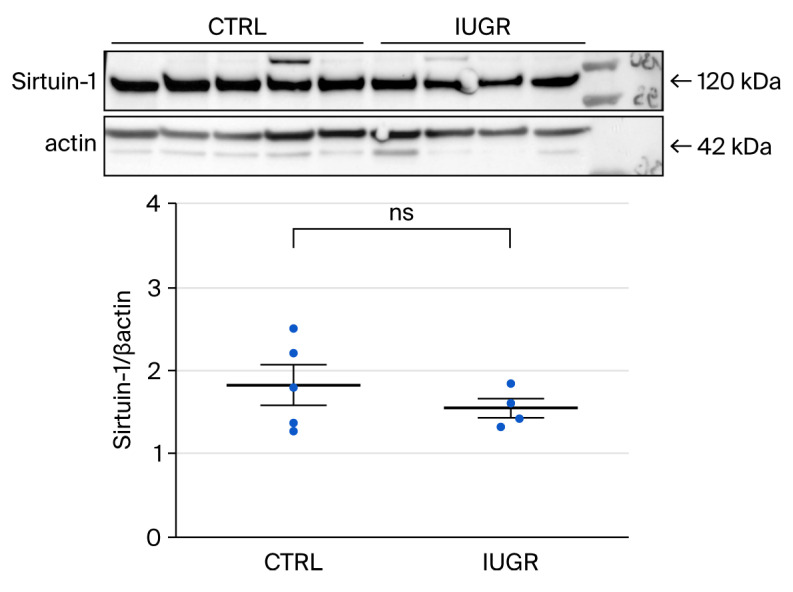
Sirtuin-1 expression measured by Western blot in ECFCs isolated from six-month-old female CTRL (n = 5) and IUGR (n = 4) rats. ns: not significant.

Western blot revealed no significant difference in Sirtuin-1 expression between ECFCs from CTRL and IUGR rats (CTRL (n = 5) vs. IUGR (n = 4) (mean (A.U.) ± SEM): 1.82 ± 0.42 vs. 1.54 ± 0.17; *p* > 0.05) (Figure 11).

## 4. Discussion

The findings of this study confirm that compared to CTRL, IUGR females exhibited no vascular alterations, as indicated by the absence of differences in PWV and an improvement in arterial elasticity, characterized by an increase in elastin fiber and a decrease in collagen fiber density. In addition, compared to CTRL-ECFCs, our results show that ECFCs from six-month-old female rats exposed to IUGR retain their overall endothelial identity (CD31^+^/CD146^+^ ratio on CD45^−^ viable cells) but display subtle alterations in progenitor marker expression (CD34). Functionally, they exhibit a shifted proliferative time course between 6 and 24 h, while their ability to form capillary-like structures remains unchanged. Regarding the NO pathway, we observed a biologically relevant trend toward reduced NO levels and decreased eNOS expression. In contrast, markers of oxidative stress and SIPS were unaffected. These results also show that the consequences of IUGR on vascular function are sexually dimorphic when compared to those we previously obtained in male rats [36] (Table 1).

Individuals born following IUGR are at increased risk of developing CVD later in life. In our laboratory, we employ a rat model of IUGR induced by maternal protein restriction and have observed that only six-month-old male offspring have increased SBP, microvascular rarefaction, and hepatic/metabolic dysfunction compared to CTRL male rats at the same age [48]. Additionally, reduced numbers of ECFCs were isolated from the bone marrow of these male animals compared to control animals, and their function was impaired [36]. In this study, we confirmed the absence of vascular alterations in IUGR females through the assessment of vascular stiffness. Compared with CTRL, IUGR females showed no significant differences in PWV, which is the current standard for measuring in vivo vascular stiffness. Similar result concerning the absence of difference in PWV has been observed in female rats exposed to fructose and high salt diet compared to a CTRL diet [49]. PWV is a global functional marker of arterial stiffness influenced by both arterial wall structure and hemodynamic factors such as blood pressure, vascular tone, and smooth muscle contractility. In young adult female rats, these functional determinants may overshadow moderate structural changes, limiting the sensitivity of PWV to early or compensatory remodeling [50]. In IUGR females, the observed increase in elastin and decrease in collagen fiber density in the aorta suggests adaptive remodeling promoting compliance. Additionally, we measured the PWV over a long arterial segment (carotid-femoral), while matrix analyses focused on the thoracic aorta, so localized changes may not affect overall PWV [51]. Also, sex-specific protective mechanisms, possibly related to female [52] hormones, may help preserve arterial function, yielding similar PWV values despite differences in aortic composition [53].

Concerning ECFCs, we previously observed in IUGR males reduced numbers and impaired functionalities compared to CTRL males [36]. However, it remains unclear whether age-matched female from the same litter counterparts experience similar ECFC alterations. Historically in preclinical studies, female animals have been underrepresented partly due to concerns that hormonal fluctuations associated with the estrous cycle may increase inter-individual variability [54], although it has been shown that co-housing female rats can synchronize their estrous cycles, thereby reducing this variability [52]. Importantly, sex is now recognized as a critical biological variable that can influence the development and progression of CVD at multiple levels [55].

We isolated ECFCs expressing surface markers such as CD31^+^, CD146^+^, and CD34^+^ and CD45^−^ [56,57] from six-month-old CTRL and IUGR female rats. CD34 is a surface antigen broadly expressed in stem and progenitor cells, including EPCs [58]. It is well established that this cellular marker enables the phenotyping of EPCs that incorporate into ischemic zones and contribute to vasculogenesis [42,59]. Flow cytometry analysis of ECFC surface markers indicates that the overall endothelial identity (assessed as the CD31^+^/CD146^+^ ratio on CD45^−^ viable cells) is preserved in IUGR-ECFCs with no significant difference compared with CTRL suggesting that IUGR does not alter the general endothelial lineage composition of circulating ECFCs, contrary to males [36]. Similarly, exposure to ambient fine particulate matter, a key component of air pollution associated with increased reactive oxygen species (ROS) production, in C57BL/6 mice (8–10 weeks) selectively decreased the circulating EPC population in male mice with no significant impact on circulating EPCs in female mice [60].

In contrast, CD34 expression showed a trend toward reduction in IUGR-ECFCs. While the difference did not reach significance in a two-sided test (*p* = 0.079), and given the reduction in CD34 expression observed in our previous study [36] conducted on IUGR male rats, we also expected to see a reduction in expression in IUGR female rats. Therefore, using a more powerful one-tailed test, we observed a one-sided analysis assuming a directional hypothesis reached significance (*p* = 0.039), suggesting that IUGR-ECFCs may exhibit subtle changes in progenitor marker expression, potentially reflecting altered maturation or functional capacity, which could have implications for their regenerative or angiogenic potential.

ECFC function was assessed by measuring their ability to proliferate and to form capillary-like structures, a process that was impaired in ECFCs from male IUGR rats [36]. The proliferation assay indicates a shifted time-course of proliferative capacity in ECFCs derived from IUGR rats compared to controls. At 6 h, IUGR-ECFCs show reduced DNA synthesis, suggesting impaired or delayed entry into the cell cycle. Whereas at 24 h, IUGR-ECFCs outperform CTRL-ECFCs in terms of proliferation, suggesting a compensatory or delayed proliferative response.

When seeded on Matrigel, ECFCs from both CTRL and IUGR females formed inter-connected pseudo-tubular networks with comparable organization and morphology, supporting the notion of a preserved angiogenic potential in IUGR females. Similar observation had been performed in populations with coronary artery disease and in healthy groups [61]. The NO pathway plays a critical role in modulating EPC function [62]. In particular, NO is essential for angiogenesis [63], as it is involved in the mobilization of ECFCs and enhances their migratory and proliferative activities [64], thereby improving endothelial repair capacity by regulating angiogenic activity [65]. So, Impaired NO production markedly diminishes these cellular [66]. In the vasculature, NO is generated by eNOS during the degradation of L-arginine to L-citrulline in the presence of molecular oxygen and nicotinamide adenine dinucleotide phosphate [67,68]. In IUGR females, we anticipated a reduction in NO production and eNOS expression compared with CTRL, similar to what was previously observed in age-matched IUGR males from the same litter [36]. Although no statistically significant difference was detected, analysis of individual DAF-2DA fluorescence values revealed distinct response trajectories between IUGR and CTRL females. Specifically, under basal conditions and following acetylcholine stimulation, IUGR females exhibited lower fluorescence intensity than CTRL. Furthermore, acetylcholine stimulation increased fluorescence intensity in CTRL females, whereas this response was minimal in IUGR females, with fluorescence levels remaining largely unchanged. Regarding eNOS expression, no significant difference was observed using a two-sided test (*p* = 0.106). However, given our a priori hypothesis of reduced eNOS expression in IUGR females—consistent with findings in age-matched IUGR males from the same litter [36], a one-sided test revealed a trend toward lower eNOS expression in IUGR-ECFCs that approached significance (*p* = 0.053). Together, these results indicate reduced NO bioavailability in IUGR-ECFCs, potentially attributable to decreased eNOS expression, and suggest early endothelial dysfunction consistent with observations previously reported in six-month-old IUGR males [36].

This impairment of NO production may be related to the decreased CD34 fluorescence intensity observed in IUGR-ECFCs, as observed in male rats of the same age and litter [36]. However, the delay in endothelial dysfunction that we observed in IUGR females compared to IUGR males may be explained in part by the cardioprotective roles of estrogens, particularly estradiol, which can promote vasodilation and inhibit fibrosis and hypertrophy [69]. Indeed, estrogens can enhance EPC mobilization, survival, migration, and endothelial differentiation and stimulate angiogenesis through binding to estrogen receptors α and β on endothelial cells, improving vascular endothelial growth factor transcription and increasing NO release via eNOS activation [70,71,72,73].

In addition to NO, oxidative stress and cellular senescence can also modulate ECFC function. Under normal physiological conditions, low levels of ROS are essential to regulate cellular responses through signal transduction and transcriptional pathways. However, when ROS production becomes excessive and unregulated, it leads to a pathological state known as oxidative stress, which plays a critical role in the development and progression of numerous diseases [74,75]. ECFCs are particularly sensitive to oxidative stress [76] and depend on an effective antioxidant defense system for survival and tissue repair. Indeed, oxidative stress induced in vitro by morpholinosydnonimine hydrochloride, has been shown to impair ECFC proliferation, migration, and angiogenic capacity [66]. In contrast to IUGR-ECFCs from males [36], there were no differences in superoxide anion production in ECFCs from CTRL and IUGR females at six months. Similar observations have been reported for EPCs [60] and other vascular cells [77,78] from animals exposed to ambient fine particles, in which intracellular ROS production was higher in exposed males than in CTRL animals, but there were no differences in females. Regarding antioxidant defenses, we observed no differences in Cu/Zn SOD1 or catalase expression between ECFCs from six-month-old IUGR and CTRL females, which may be explained by the absence of increased ROS production.

We also measured the protein expression of Sirtuin-1, the most studied member of the mammalian Sirtuin family. Sirtuin-1 is a key molecule in maintaining redox homeostasis [79], levels and increasing antioxidant defenses [80] notably by reducing ROS level.

Unlike ECFCs from IUGR males, in which Sirtuin-1 expression was reduced compared to CTRL, there were no differences between ECFCs from six-month-old CTRL and IUGR females. This is not surprising, as there was no difference in ROS production or antioxidant defenses. These data show that, compared to six-month-old IUGR males [36], ECFCs from age-matched IUGR females do not have oxidative stress markers, a trend also observed in healthy individuals, with women generally having lower levels of oxidative stress markers than men [81]. Indeed, there is considerable evidence in the literature that defense against ROS is more proficient in females than in males [74]. Estrogens, by virtue of their chemical structure, have a strong antioxidant action [82] and can increase glutathione levels [83,84] and specific enzymes involved in antioxidant defense, such as SOD [84]. Several studies have also shown the involvement of estrogens in the generation of EPCs, their mobilization from bone marrow, and their incorporation into ischemic areas [73,85,86], which could partly explain their vascular protective effects, although direct hormonal measurements (e.g., serum estradiol) were not available in the present study and represent a limitation.

In addition to maintaining redox balance, Sirtuin-1 has been shown to protect endothelial cells from SIPS [87]. In growth-restricted newborns, Vassalo et al. demonstrated that decreased Sirtuin-1 expression accelerates ECFC aging and acts as a determining factor in ECFC angiogenic defect [29]. To assess cellular senescence, we measured β-gal activity, the most widely used biomarker of senescence [88]. There was no difference in β-gal activity in ECFCs from CTRL and IUGR females at 6 months, in contrast to male animals [36]. This finding may be related to the lack of difference in Sirtuin-1 expression mentioned earlier which could contribute to delayed cellular senescence [29]. This lack of markers of senescence is consistent with previous studies suggesting that cellular senescence may contribute to sex-specific differences in the development and progression of various pathologies [89,90,91]. Since senescence is involved in tissue dysfunction and disease, it is plausible that its regulation is sexually dimorphic. Such differential regulation may result in sex-specific accumulation of senescent cells, thereby contributing to distinct clinical phenotypes. Supporting this hypothesis, previous research has demonstrated that male mice have a greater burden of senescent cells than females, which persists throughout their lifespan [92].

## 5. Conclusions

**Figure 12:**
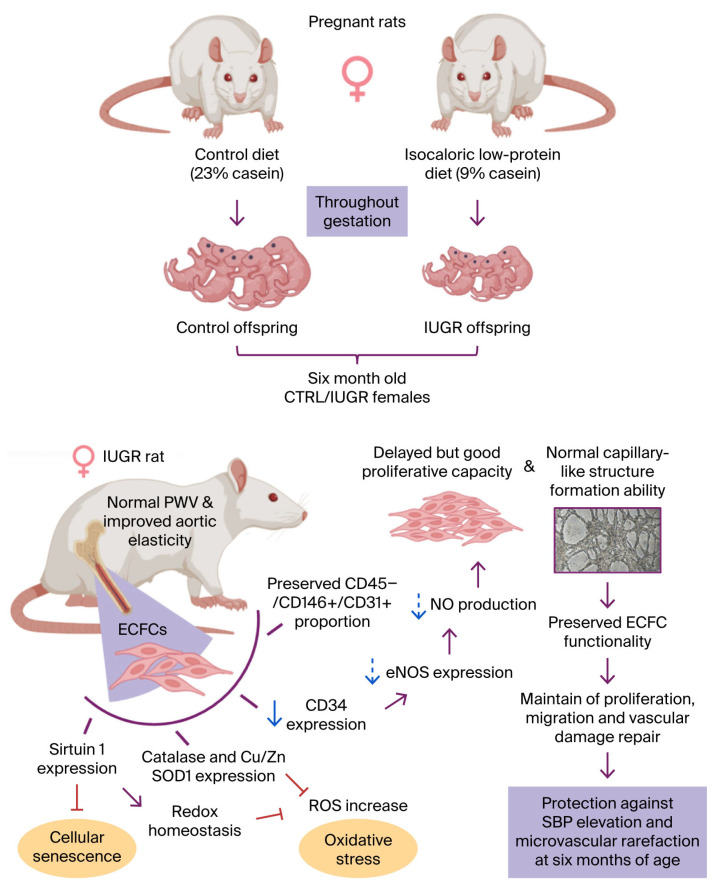
Summary of functionally preserved endothelial colony-forming cells (ECFCs) in our rat model of intra-uterine growth restriction (IUGR). Pregnant rats were assigned to a control or isocaloric low-protein diet, giving birth to control (CTRL) and IUGR pups, respectively. At six months of age, IUGR females displayed no change in pulse wave velocity (PWV) but improvement of aortic elasticity, compared to CTRL females. ECFCs were isolated from the bone marrow of females from both groups; the proportion of CD45^−^, CD146^+^, and CD31^+^ expressing cells was the same in the two groups. ECFCs from IUGR rats showed decreased CD34 expression, with a tendency towards reduced endothelial nitric oxide synthase (eNOS) expression and nitric oxide (NO) production compared to CTRL rats. This slight decrease observed in IUGR-ECFCs could explain their initially delayed proliferative capacity, which was nevertheless compensated for over time, with the same ability to form capillary-like structures as CTRL-ECFCs. ECFCs from CTRL and IUGR rats also expressed Sirtuin 1, catalase, and Cu/Zn superoxide dismutase 1 (SOD1) to similar degrees, preventing cellular senescence and oxidative stress by blocking the increase in reactive oxygen species (ROS). Preservation of ECFC functionality may maintain vascular health, potentially protecting female IUGR rats from elevated systolic blood pressure (SBP) and microvascular rarefaction. Violet arrows: leading to. Red arrows: inhibiting. Blue arrow: decrease. Blue dotted arrows: downward trend. Created using a licensed version of BioRender.com.

In summary, compared to males from the same age and litter [36], ECFCs from six-month-old female rats exposed to IUGR maintain their endothelial identity, with only subtle alterations in progenitor marker expression. Although they show a transient shift in proliferative dynamics, their capacity for capillary-like network formation is preserved. A trend toward reduced NO production and decreased eNOS expression suggests a potential vulnerability in endothelial function, yet markers of oxidative stress and stress-induced premature senescence remain unaffected. Overall, these findings indicate that at six months of age, IUGR-ECFCs exhibit only minor changes, which may contribute to maintaining protection against arterial hypertension, microvascular rarefaction and arterial stiffness (Table 1; Figure 12).

## 6. Limitations

This study has several limitations. First, the number of animals used in this study was relatively small, primarily because of the fragility of ECFCs, which are particularly difficult to isolate at six months of age. In several cases, ECFC cultures failed due to cell fragility or occasional contamination. Although these failures occurred in both groups without a discernible pattern, we cannot completely rule out the possibility of a subtle selection bias. Another limitation is the lack of analysis across both sexes within the same experiments, which would have helped reduce experimental and experimenter-related biases.

Considering the sexual dimorphism observed in our adult IUGR rat model, future studies should explore the potential protective role of estrogens in preserving ECFC functionality. One limitation of our study is the lack of direct hormonal measurements (e.g., serum estradiol). Consequently, the proposed vascular protective effect of estrogens remains speculative. Future investigations incorporating hormonal assessments will be essential to more robustly evaluate this hypothesis.

## 7. Translational Perspective

ECFCs are critical for vascular repair, but aging is associated with reduced ECFC number and function due to increased oxidative stress, cellular senescence, and impaired NO signaling, contributing to endothelial dysfunction. In females, estrogens enhance ECFC survival and activity (via eNOS and PI3K/Akt pathways); menopause-related estrogen decline is therefore associated with ECFC dysfunction and loss of cardiovascular protection [73,93].

In our study, 6-month-old IUGR females showed no overt vascular alterations and only mild ECFC changes. However, we hypothesize that with aging, notably at 12 months of age, these protective features may decline, leading to vascular dysfunction associated with impaired ECFC number and function, similarly to those observed in IUGR males [36].

Clinically, it would be of interest to identify early ECFC-related biomarkers, particularly epigenetic factors, which may help identify individuals with IUGR who are at increased cardiovascular risk prior to disease onset. Moreover, strategies aimed at preserving ECFC function—such as lifestyle interventions, pharmacological approaches, or cell-based therapies factors—may represent promising preventive avenues [94,95].

## Figures and Tables

**Table 1:** Sexual dimorphism in IUGR rats. Table showing the differences observed between six-month-old male and female IUGR rats in terms of vascular function and ECFC. Individuals of both sexes came from the same litters but were studied and compared with CTRL individuals separately, with males being studied before females [36]. Overall, IUGR males have impaired ECFC/vascular function, whereas this function is generally preserved in IUGR females. ECFCs: endothelial colony-forming cells, eNOS: endothelial nitric oxide synthase, IUGR: intrauterine growth restricted, NO: nitric oxide, SIPS: stress-induced premature senescence.

	**IUGR Male Rats**		**IUGR Female Rats**
×	Elevated systolic blood pressure.	√	Normal systolic blood pressure.
×	Microvascular rarefaction.	√	Absence of microvascular rarefaction.
	**ECFCs of IUGR Male Rats**		**ECFCs of IUGR Female Rats**
×	Reduction of total number.	×	Slight alterations in CD34 expression.
×	Alteration in proliferation capacity.	√	Delay in proliferation phase between 6 h and 24 h.
×	Altered ability to form capillary-like structures.	√	Preserved ability to form capillary-like structures.
×	Reduction in NO production.	×	Slight reduction in NO levels and eNOS expression.
×	Presence of oxidative stress and SIPS markers.	√	Absence of oxidative stress and SIPS markers.

## Data Availability

All results are included in this study.

## Abbreviations

The following abbreviations are used in this manuscript:

AHT	Arterial hypertension
BrdU	5′-Bromo-2′-deoxyuridine
CTRL	Control
DAF-2DA	4,5-Diaminofluorescein diacetate
DHE	Dihydroethidine
EBM2	Endothelial basal cell growth culture medium-2
ECFCs	Endothelial colony-forming cells
ECG	Electrocardiography
eNOS	Endothelial nitric oxide synthase
EPCs	Endothelial progenitor cells
FCS	Fetal calf serum
HEPES	4-(2-Hydroxyethyl)-1-piperazineethanesulfonic acid
IUGR	Intrauterine growth restriction
MNCs	Mononuclear cells
MV2	Endothelial cell growth medium
NO	Nitric oxide
PBS	Phosphate-buffered saline
PWV	Pulse wave velocity
ROS	Reactive oxygen species
SBP	Systolic blood pressure
SIPS	Stress-induced premature senescence
SOD1	Cu/Zn Superoxide dismutase
VEGFR-2	Vascular endothelial growth factor receptor-2
